# Efficient crystal structure materials as reactive sorbent for the CO_2_ and CH_4_ adsorption and storage

**DOI:** 10.1038/s41598-024-57060-8

**Published:** 2024-03-19

**Authors:** R. Essehli, B. Aïssa, T. Altamash, M. Lachkar, M. Atilhan, B. El Bali, G. R. Berdiyorov, A. Amhamed

**Affiliations:** 1 Energy and Transportation Science Division, Oak Ridge National Laboratory (ORNL), 1 Bethel Valley Rd, Oak Ridge, TN 37830 USA; 2 Qatar Environment and Energy Research Institute (QEERI), Hamad Bin Khalifa University (HBKU), Qatar Foundation, P.O. Box 34110, Doha, Qatar; 3 Materials Science, Energy and Nanoengineering Department (MSN), Mohammed VI Polytechnic University (UM6P), Lot 660 – Hay Moulay Rachid, 43150 Ben Guerir, Morocco; 4 University Sidi Mohamed Ben Abdellah, Fez City, Morocco; 5 Department of Chemical and Paper Engineering, Western Michigan University, Floyd Hall, A-230, Kalamazoo, MI 49008 USA; 6 Laboratory of Mineral Solid and Analytical Chemistry, “LMSAC”, Department of Chemistry, Faculty of Sciences, University Mohamed I, Po. Box 717, 60000 Oujda, Morocco

**Keywords:** Environmental sciences, Environmental impact, Chemical engineering, Chemical synthesis, Synthetic chemistry methodology

## Abstract

The efficient dirubidium cobalt bis(dihydrogendiphosphate) dihydrate compound is successfully synthesized in a solution and used as a reactive sorbent for the CO_2_ and CH_4_ gases adsorption and storage. A crystal of this Rb_2_Co(H_2_P_2_O_7_)_2_·2H_2_O compound has been isolated and characterized by single X-ray diffraction analysis and was found to crystallize in the triclinic system ($P\overline{1}$) with the cell parameters (Å): 6.980(1), 7.370(1), 7.816(1), 81.74(1), 70.35(1), 86.34(1); V = 374.68(9) Å3, Z = 2. The crystal-packing consists of a three-dimensional framework made upon corners and edges sharing of [RbO_7_], [H_2_P_2_O_7_] and [CoO_6_] entities, furthermore linked by a network of H-bonds. The UV–Vis spectroscopy revealed usual transitions between the ground state 4T1g and the upper levels 4T2g, 4A2g and 4T1g (P). Moreover, the CO_2_ and CH_4_ gases sorption measurements were successfully performed at two different temperatures (25 and 45 °C) and various pressures ranging from vacuum to 50 bar. Our results show that rate of CO_2_ and CH_4_ capturing was 3.10 mmol/g and 2.35 mmol/g at temperature 25 °C and pressure 50 bar, respectively. This compound showed a clear potential for CO_2_/CH_4_ adsorption and storage thereby paving the way towards its exploration and adaptation for capturing and collecting carbon dioxide and greenhouse gases from the air, and their conversion into hydrocarbon fuels using existing mature technologies. We have also conducted density functional theory calculations to study the CO_2_ and CH_4_ adsorption properties of Rb_2_Co(H_2_P_2_O_7_)_2_·2H_2_O. The simulation results show enhanced adsorption of both types of molecules on the surface of the material.

## Introduction

Climate change is a critical world pressing challenge. Emission of greenhouse gases, including nitrous oxide (N_2_O), methane (CH_4_) and carbon dioxide (CO_2_), have increased global temperatures by around 1 °C since pre-industrial times, where CO_2_ alone is contributing to more than 60% of this global warming^1–3^. The increasing percentage of CO_2_ and CH_4_ gases into the atmosphere is considered nowadays as a serious threat as it has a range of potential ecological, physical and health impacts, including extreme weather events (such as floods, droughts, storms), sea-level rise, altered crop growth, and disrupted water systems. To mitigate climate change, UN member parties have set a target, in the Paris Agreement, of limiting average warming to 2 °C above pre-industrial temperatures.

From technical viewpoint, the absorption and adsorption of greenhouse gases is one of the most challenging technological issues for researchers and environmentalist towards developing sustainable technologies based on new materials to control and mitigate the anthropogenic emission^1^. For more than six decades, solvent absorption technologies, and particularly aqueous alkanolamines, were the most commonly employed chemical absorbents for CO_2_ gas removal.

However, these adsorption processes are associated with various industrial limitations^1,4^. In the recent development in the chemical sciences field, ionic liquids (ILs) have shown some potential features for CO_2_ capturing due to their unique chemical and physical properties and replacement capability of serious conventional solvents. In addition, deep eutectic solvents (DESs) have demonstrated many unusual characteristics for CO_2_ absorption at room temperature^5–9^, including negligible vapor pressure, non-flammability, wide liquid range, high thermal and chemical stabilities and high solvation capacity^10,11^. DES term was introduced by the team of Abbott^9^ in 2003 in order to overcome some of the IL’s drawbacks. Similarly, dried solid materials are another type rational choice for gas adsorption in separation technologies which helps to minimize the energy consumption and regeneration processes up to some extent. Thus, a number of newer solid materials are now being synthesized and characterized and tuned further in order to enhance their practical applicability for specific gas or gas mixtures^12–15^. Indeed, gas adsorptivity capacity is closely related to accessible surface structure and molecular arrangement of the solid crystal. Typically, the flue gas composition consists of CO_2_, H_2_O, CO, N_2_, H_2_S, H_2_, Ar, NO_x_, SO_x_, Hg and Cd^16^. In some cases, CH_4_ slip into flue gas stream could occur when the incomplete exothermic oxidation of a fuel took place due to insufficient amount of oxygen to the combustion system. R&D tends to focus on safe and eco-friendly porous organic and inorganic materials for their use in gas storage^17–20^. Since then, several inorganic phosphate-based compounds have been extensively studied and were found to not only be able to capture CO_2_ and CH_4_ but also to act as storage for clean energy. As a contribution to this internationally critical research effort, we successfully designed and synthesized efficient diphosphates, namely dirubidium cobalt bis(dihydrogendiphosphate) dihydrate (Rb_2_Co(H_2_P_2_O_7_)_2_·2H_2_O), and assessed it as CO_2_ and CH_4_ gases adsorption and storage. Our results show a rate of CO_2_ and CH_4_ capturing of 3.10 mmol/g and 2.35 mmol/g at temperature of 25 °C and pressure of 50 bar. Moreover, gas sorption comparison study demonstrated a clear evidence of a selective behavior of our compound for CO_2_ and CH_4_ gases at each temperature and pressure, and revealed that adsorbtivity of CO_2_ is more than that of the CH_4_ at different temperatures and pressures, making this diphosphate a promising candidate for greenhouse gases adsorption and storage. First principles density functional theory (DFT) calculations also reveal strong adsorption of both CO_2_ and CH_4_ molecules on the surface of the Rb_2_Co(H_2_P_2_O_7_)_2_·2H_2_O material. The adsorption energy of CO_2_ molecules is smaller (i.e., stronger adsorption) as compared to the adsorption energy of CH_4_ molecule due to covalent character of bonding of the CO_2_ molecule on the surface of the material as revealed in our electronic structure calculations.

The present work focuses on designing a diphosphate that can be used for direct air capture of CO_2_ and CH_4_ molecules. This work could lead to promising technology to create CO_2_ and CH_4_ outlet from the earth’s atmosphere due to several advantages such as the easy application and geographical independence. The present new configuration can also help bypassing the high cost of DAC systems for greenhouse gas capture.

## Methods

### Sample fabrication

Single crystals of Rb_2_Co(H_2_P_2_O_7_)_2_·2H_2_O was prepared according to the method reported in our previous works^21–26^. K_4_P_2_O_7_ (1 mmol), Rb_2_CO_3_ (1 mmol), and CoCl_2.6_H_2_O (0.5 mmol), dissolved in a few ml of diluted HCl (0.75 mL of HCl 0.1 M), as structure directing agent. The mixture was stirred for 2 h. The evaporation of the solvent water was allowed for one week, at room temperature. Prismatic crystals with edge-length up to 0.2 mm was deposited at the end of this period, then filtered-off and washed with water–ethanol mixture (concentration 20:80).

### Characterization

The reported crystallographic data for Rb_2_Co(H_2_P_2_O_7_)_2_·2H_2_O are given in Table 1. The Rb_2_Co(H_2_P_2_O_7_)_2_·2H_2_O selected crystal shows an excellent structural quality with exceptional low Rint factors. The hydrogen atoms are localized in different Fourier maps. They are also refined independently using solely the condition that the temperature parameters of hydrogen atoms in H_2_O molecules and H_2_P_2_O_7_ groups are equal. X-ray diffraction data were collected on an Oxford Diffraction XCALIBUR four-circles X-ray diffractometer using graphite monochromatized MoKα radiation (λ = 0.7173 Å) equipped with a SAPPHIR CCD two-dimensional detector. The intensity data were corrected for Lorentz and polarization effects. A numeric analytical absorption correction was carried out with the program CrysAlis RED^27^. The metal and phosphorus atoms were located by direct methods, using the SHELXS-97 program^28^, while the remaining atoms were found from successive Fourier difference maps. Atomic positions were refined by full matrix least-squares method using SHELXL-97 program^29^. Full-matrix least squares refinement was based on F2. Thermal displacements of all non-hydrogen atoms were refined anisotropically. Pertinent crystallographic details are given in Tables 1, 2 and 3. Graphics have been performed using DIAMOND program^30^. Tables of crystal structures and refinements, notably full bond lengths and angles, and anisotropic thermal parameters have been deposited with the Inorganic Crystal Structure Database, FIZ, Hermann von Helmholtz Platz 1, 76,344 Eggenstein Leopoldshafen, Germany; fax: (+ 49) 7247 808 132; Email: crysdata@fiz-karlsruhe.de. CSD-deposition numbers are respectively 421807 for Rb_2_Co(H_2_P_2_O_7_)_2_·2H_2_O.

**Table 1:** Crystal data and structure refinement for Rb_2_Co(H_2_P_2_O_7_)_2_·2H_2_O.

Chemical formula	Rb_2_Co(H_2_P_2_O_7_)_2_·2H_2_O
Formula weight	387.9 (g/mol)
Colour	Pink
Symmetry (S.G.)	Triclinic (P-1)
Unit cell parameters (Å, °) a/b/c/α/β/γ	6.980(1) 7.370(1) 7.816(1) 81.74(1) 70.35(1) 86.34(1)
V (Å^3^)	374.68(9)
Z	2
ρ_calc._ (g cm^−3^)	2.738
Temperature (K)	302(2)
Diffractometer	Oxford Diffraction CCD
Radiation MoKα, (Å)	0.71069
2*θ*-range	2.842–27.679
Reciprocal space	− 8 ≤ h ≤ 8 − 8 ≤ k ≤ 9 − 9 ≤ l ≤ 9
Collected reflections	1508
Refined parameters	118
R(F)/wR2(F^2^)	0.0340/0.0851

**Table 2:** Atomic coordinates and isotropic displacement parameters U_eq_^a^ for Rb_2_Co(H_2_P_2_O_7_)_2_·2H_2_O.

Atom	*Site*	x	y	z	Ueq
Co1	1c	0.0000	0.5000	0.0000	0.0152 (2)
P1	2i	0.26553 (15)	0.20005 (14)	− 0.25549 (14)	0.0153 (2)
P2	2i	− 0.14918 (16)	0.26198 (13)	− 0.25914 (14)	0.0158 (2)
O1	2i	0.2558 (4)	0.3480 (4)	− 0.1401 (4)	0.0231 (7)
O2	2i	0.4544 (4)	0.2162 (4)	− 0.4320 (4)	0.0261 (7)
H2	2i	0.563(5)	0.266(6)	− 0.420(7)	0.031
O3	2i	0.2445 (5)	0.0076 (4)	− 0.1553 (4)	0.0275 (7)
O4	2i	0.0920 (4)	0.2310 (4)	− 0.3509 (4)	0.0225 (6)
O5	2i	− 0.1883 (4)	0.3833 (4)	− 0.1114 (4)	0.0197 (6)
O6	2i	− 0.2239 (4)	0.3339 (4)	− 0.4151 (4)	0.0220 (6)
O7	2i	− 0.2412 (5)	0.0693 (4)	− 0.1731 (4)	0.0259 (7)
H7	2i	− 0.234(8)	0.039(6)	− 0.060(3)	0.031
O8	2i	0.0601 (5)	0.7123 (4)	− 0.2196 (4)	0.0262 (7)
H8A	2i	0.104(6)	0.693(6)	− 0.331(2)	0.031
H8B	2i	0.126(6)	0.803(4)	− 0.213(5)	0.031
Rb1	2i	− 0.40751 (7)	0.71568 (6)	− 0.21039 (6)	0.03062 (18)

**Table 3:** Bond lengths (Ǻ) values in the structures of Rb_2_Co(H_2_P_2_O_7_)_2_·2H_2_O.

(1)	
Atoms 1,2	d 1,2* [Å]
Co1—O5	2.087(3)
Co1—O5^i^	2.087(3)
Co1—O8	2.097(3)
Co1—O8^i^	2.097(3)
Co1—O1^i^	2.103(3)
Co1—O1	2.103(3)
P1—O1	1.496(3)
P1—O3	1.509(3)
P1—O2	1.551(3)
P1—O4	1.612(3)
P2—O6	1.498(3)
P2—O5	1.503(3)
P2—O7	1.559(3)
P2—O4	1.608(3)
Rb1—O2^iv^	2.892(3)
Rb1—O7^vii^	3.010(3)
Rb1—O3^viii^	3.100(3)
Rb1—O5^iii^	3.127(3)
Rb1—O1^i^	3.219(3)
Rb1—O4^iv^	3.377(3)
Rb1—O1^ix^	3.562

*Symmetry codesin (1) and (2):

(i) − x, 1 − y, − z; (ii) 1 + x, y, z; (iii) − 1 − x, 1 − y, − z; (iv) − x, 1 − y, − 1 − z; (v) 1 + x, − 1 + y, z; (vi) x, − 1 + y, z; (vii) x, 1 + y, z; (viii) − 1 + x, 1 + y, z; (ix) − 1 + x, y, z

in (**3**): (i) − x, 1 − y, − z; (ii) − x, 1 − y, − 1 − z; (iii) − 1 − x, 1 − y, − z; (iv) 1 + x, − 1 + y, z; (v) x, − 1 + y, z; (vi) x, 1 + y, z; (vii) 1 − x, y − 1, z.

### Gas adsorption/sorption device

The absorption/desorption measurements of carbon dioxide (CO_2_) and methane (CH_4_) with the synthesized material were successfully performed at temperatures of 298 and 318 K, and from vacuum to 50 bars, by using the specific high pressure “magnetic suspension sorption apparatus (MSA)” equipment, purchased from Rubotherm Präzisionsmesstechnik GmbH. This tool has the ability to raise the pressure up to 350 bars, and temperature up to 373 °K. The pressure transducers (ParoscientificTM, USA) were installed to measure the pressure from vacuum to 350 bars with an accuracy of 0.01% bar, and temperature sensor (Minco PRT, USA) with a sensitivity of ± 0.5 °C. The detailed experimental procedure and calibration are detailed in elsewhere^31–33^.

### Density functional theory

Computer simulations are conducted using DFT within the generalized gradient approximation of Perdew–Burke–Ernzerhof (PBE) to describe the exchange–correlation energy^34^. The plane-wave basis set with a cut-off energy of 1360.57 eV is used to describe the atoms in the system. The Brillouin zone integration was conducted using 15 × 15 × 15 Monkhorst–Pack k-points sampling^35^ for the unit cell structure of the material. The convergence criterion for Hellman–Feynman forces was 0.01 eV/Å. Interactions of CO_2_ and CH_4_ molecules with the surface of the material is characterized by the electron difference density which is defined as the difference between the self-consistent valence charge density and the superposition of atomic valence densities^36^. Simulations are conducted using the computational package Atomistix toolkit^37,38^.

## Results and discussions

### Crystal structure study

**Figure 1:**
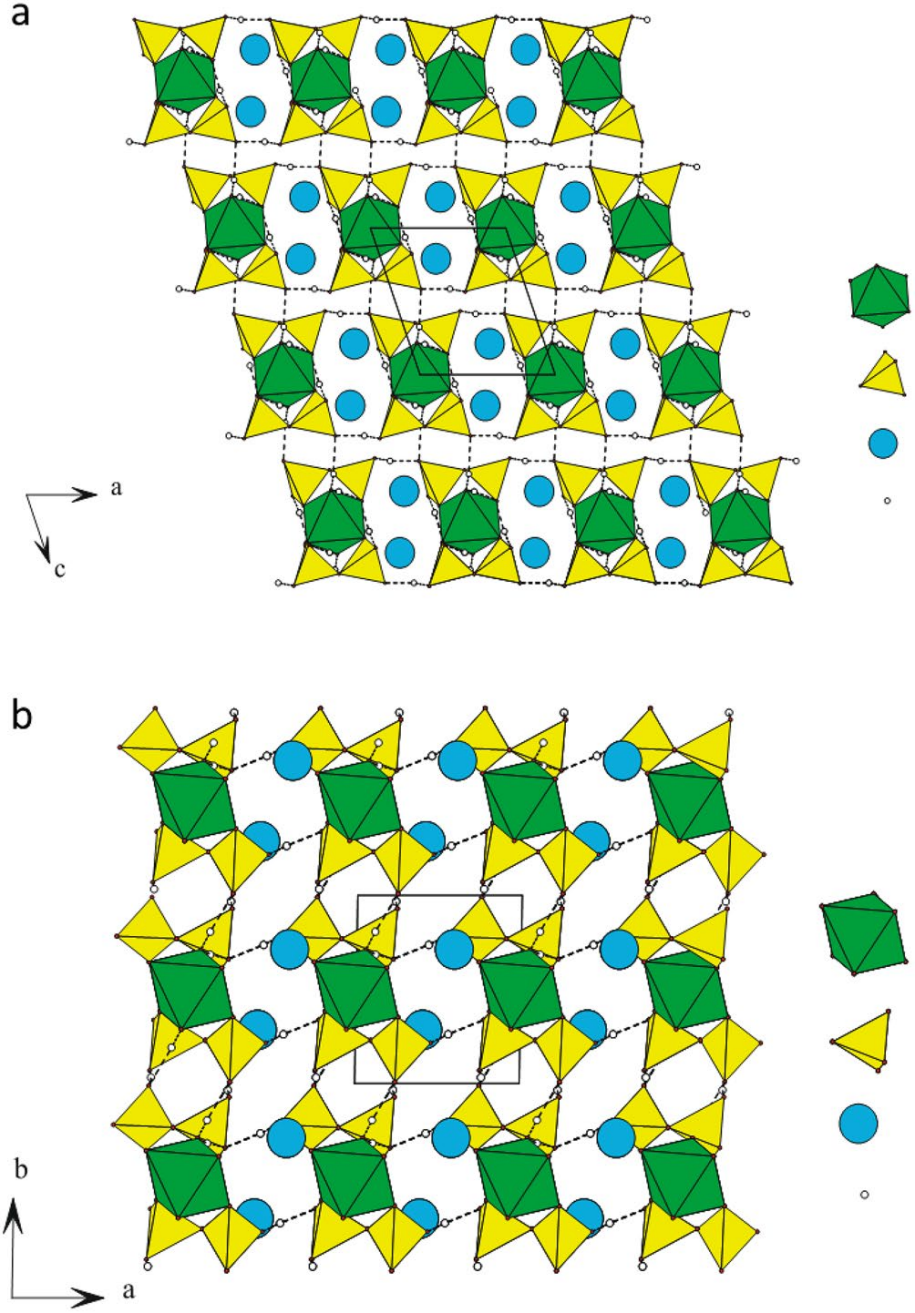
(**a**, **b**) Rb_2_Co(H_2_P_2_O_7_)_2_·2H_2_O as representative crystal structure, projections of the framework onto crystallographic (010) and (001) planes. H-bonds are represented as dashed lines.

The structural model in Rb_2_Co(H_2_P_2_O_7_)·2H_2_O consists of a 3D framework, made from corners and edges sharing of [RbO_7_], [H_2_P_2_O_7_] and [CoO_6_] entities. Figure 1a,b depict the shape of the crystal structure of the title compound onto the crystallographic (010) and (001) planes. H-bonds are represented by dashed lines. These figures have been drawn according to the respective atomic coordinates reported in Table 2 and to the bond lengths and angles represented in Table 3. The structure might be described in terms of isolated [CoO_4_(H_2_O)_2_(H_2_P_2_O_7_)_2_] units which propagates tri-dimensionally through Rb–O interactions and an intricate H-bonds network between the H_2_P_2_O_7_’s hydroxyl groups. Such unit [CoO_4_(H_2_O)_2_(H_2_P_2_O_7_)_2_] results from the edge sharing [CoO_4_(H_2_O)_2_] octahedron and [H_2_P_2_O_7_] double tetrahedrons. The unit cell contains two distinctive phosphorous (V) sites coordinated by four (04) oxygen atoms in a slightly distorted tetrahedron. Two (02) tetrahedra share an apex to form the dihydrogenpyrophosphate group H_2_P_2_O_7_. Average <P‒O> distance is about 1.551 Å. This value is similar to its homologous distances in the known isoformular compounds (NH)_2_Co(H_2_P_2_O_7_)_2_·2H_2_O^21^. As in the other mixed pyrophosphates, H_2_P_2_O_7_ shows almost an eclipsed conformation. The ∠(P–O–P) bridging angle is one feature characterizing the [H_2_P_2_O_7_]_2_- anion, here of 129.3°. In this new pyrophosphates, Co^2+^ cations are placed on inversion centre position (0, 1/2, O). Their octahedral coordination is composed of four (04) Oxygen atoms from two (02) bidendate [H_2_P_2_O_7_] groups and the remaining two (02) Oxygen from the water molecules. However, the M^2+^ cation in the orthorhombic form of K_2_M(H_2_P_2_O_7_)_2_·2H_2_O (M=Co, Ni, Cu)^22^ is located in the mirror plane within an octahedral coordination formed by two (02) bidendate [H_2_P_2_O_7_]_2_- counter ions and two (02) water molecules. Average ${\overline{\text{d}}}$(Co‒O) is of 2.095 Å, value was compared with recently the one reported value of (NH_4_)_2_Co(H_2_P_2_O_7_)_2_·2H_2_O (2.053 Å) (Table 3)^21^.

### UV–Vis study

**Figure 2:**
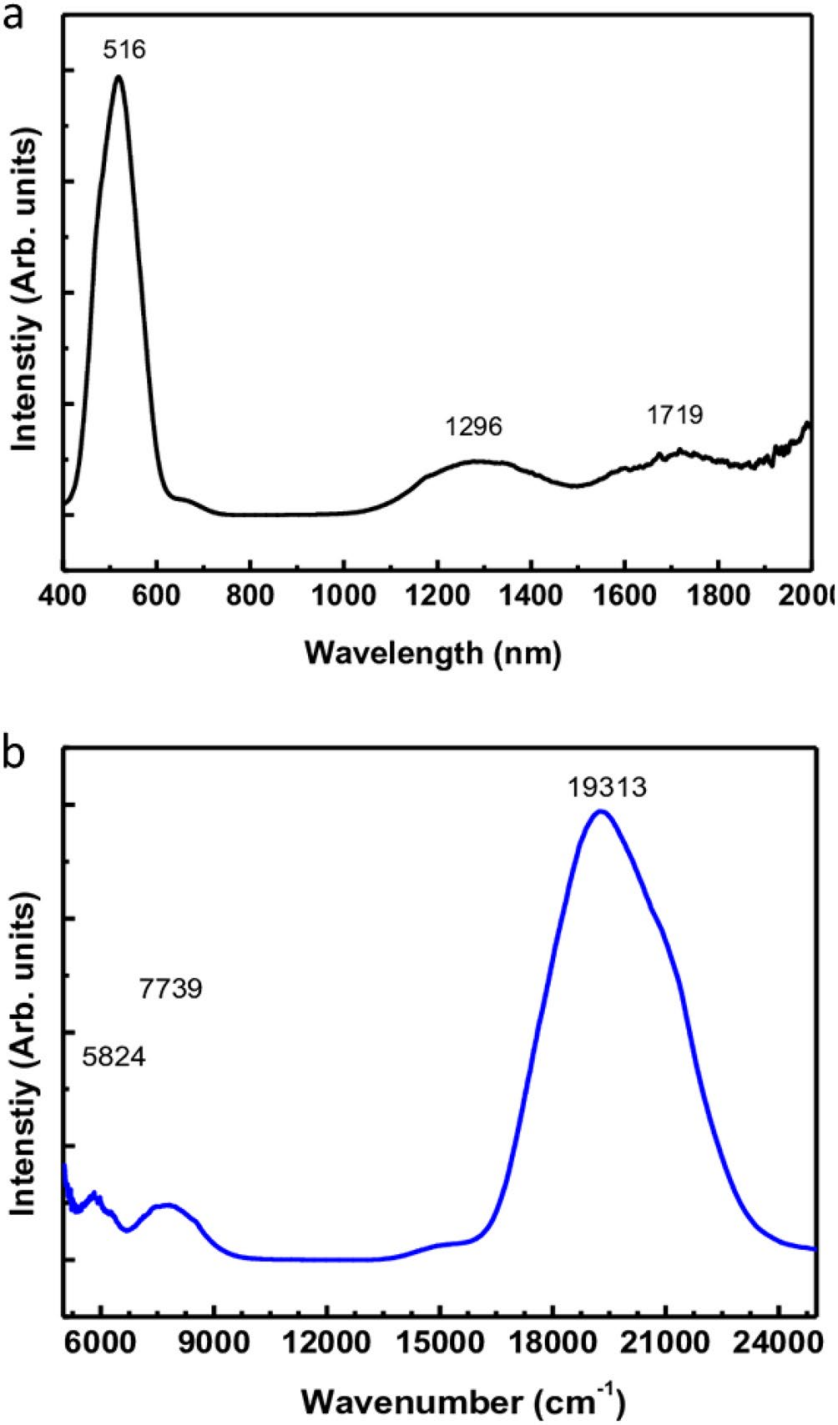
UV–Visible spectra of the Rb_2_Co(H_2_P_2_O_7_)_2_·2H_2_O compound.

The UV–Visible spectrum of the Rb_2_Co(H_2_P_2_O_7_)·2H_2_O is displayed in Fig. 2a,b. It might be described in terms of electronic transitions between the ground state 4T1g and the upper ones 4T2g, 4A2g and 4T1g(P). In fact, three spins allowed transitions may be expected. The lowest energy band observed near 7109.6 cm^−1^ is assigned to the 4T2g → 4T1g transition, while the main absorption band in the visible region near 14,581 cm^−1^ is rather assigned to 4T2g (P) → 4T1g.

### ***CO***_***2***_*** and CH***_***4***_*** adsorption and desorption***

**Figure 3:**
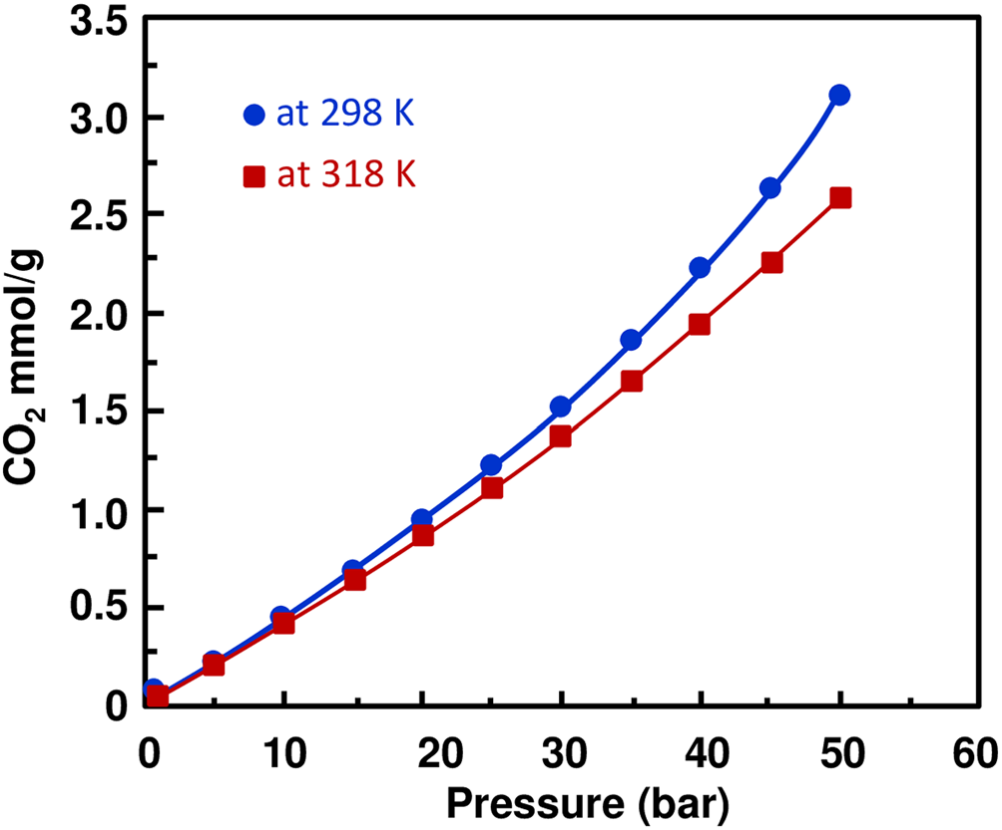
CO_2_ absorption trends in Rb_2_Co(H_2_P_2_O_7_)_2_·2H_2_O at 298 and 318 K, and different pressures.

**Figure 4:**
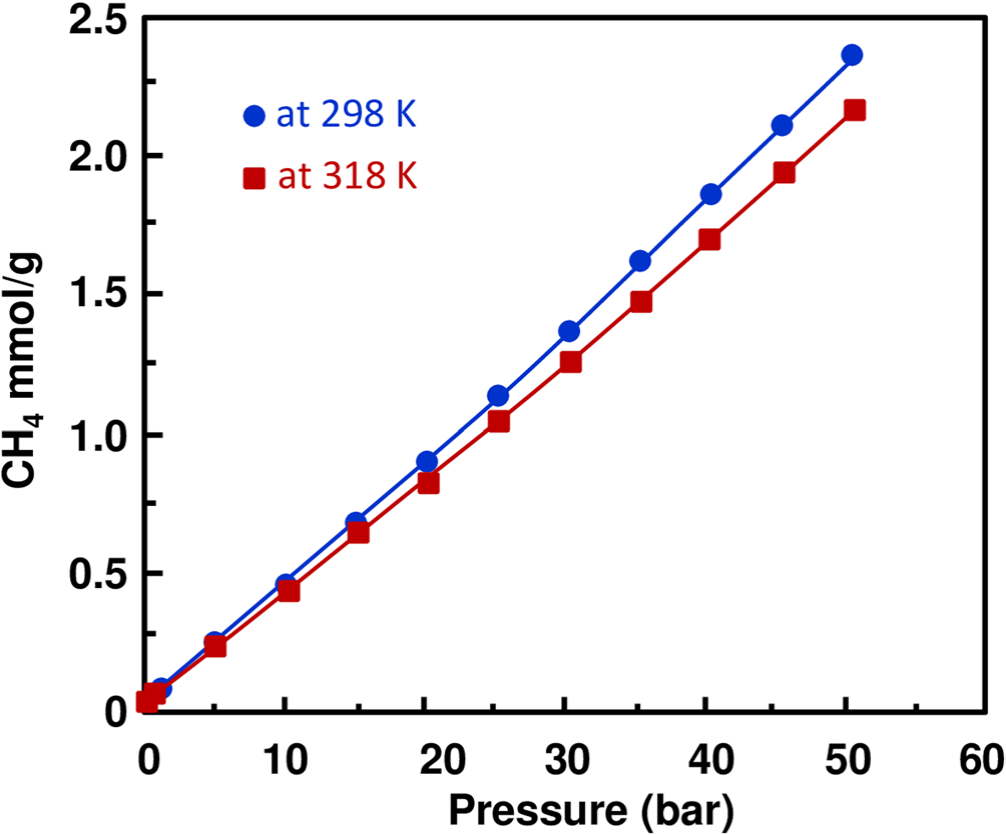
CH_4_ absorption trends in Rb_2_Co(H_2_P_2_O_7_)_2_·2H_2_O at 298 and 318 K and different pressures.

**Figure 5:**
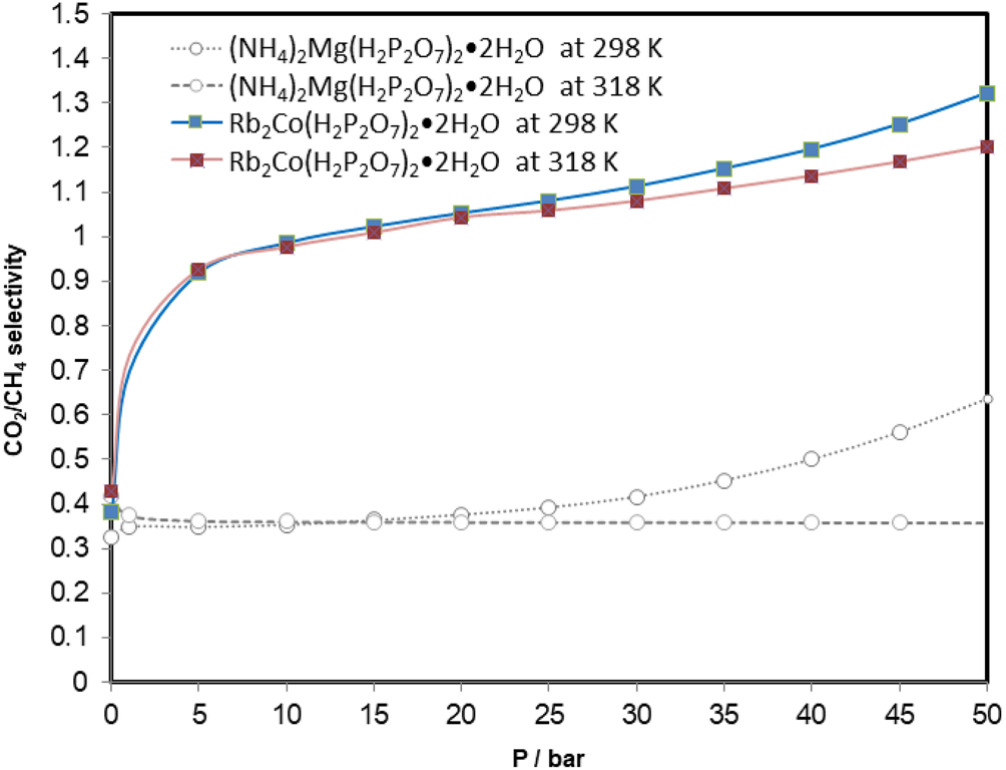
CH_4_/CO_2_ ideal selectivity at low and high pressures for studied Rb_2_Co(H_2_P_2_O_7_)_2_·2H_2_O at 298 and 318 K and different pressures.

The ultimate aim of the present work is to experimentally investigate the sorption capacity of CO_2_ and CH_4_ onto the Rb_2_Co(H_2_P_2_O_7_)·2H_2_O. For both gases, the experiments were performed at temperatures of 298 and 318 K, respectively. At each temperature, cycles of adsorption and desorption were run by stepwise increment/decrement process of CO_2_ and CH_4_ pressures, ranging from vacuum to 50 bars and its vice-versa. At each temperature, individual cycles of CO_2_ and CH_4_ were recorded that consist of 12 adsorption and 8 desorption points with Rb_2_Co(H_2_P_2_O_7_)·2H_2_O, illustrated in supplementary Tables S1 and S2. Similarly, all the values were used to plot the figures to understand the behavior (hysteresis and physisorption) of Rb_2_Co(H_2_P_2_O_7_)·2H_2_O at moderate and higher temperature from vacuum to high pressure through a programmed adsorption–desorption cycle in Figs. S1–S4. On looking over the plots (Figs. S1–S4) of their corresponding gas at both the temperature, figures demonstrate a smooth trend of variation with respect to pressure by overlapping adsorption and desorption points. These collective adsorption–desorption trends indicate that there are no hysteresis, and no significant changes occur in material. Additionally, it has to be mentioned that in order to obtain reliable data, peripheral conditions like humidity, ambient pressure, and temperature were taken care of ^39^. The similar trend of variation (adsorption and desorption pressure point that lies at same plotted line) have found in our recently published work with (NH_4_)_2_ Mg(H_2_P_2_O_7_)_2_·2H_2_O) with CO_2_ and CH_4_ at 298 and 318 K ^40^ Figs. S1, S2 and S3 show a common thermodynamic trend of variation; solubility of gas decreases when increasing the temperatures, and increases when increasing the pressures which in turns lead to increase the adsorption capacity of CO_2_ and CH_4_ in m.mol per gram of sample. Although, similar trend of adsorption/desorption indicates physisorption of gases gives the advantage of reusability for energy and economic saving ^41,42^ point of view as ionic liquids and deep eutectic solvent being tested as liquid absorbent ^33,39,42–44^. Consequently, this efficient crystal could be very useful materials for one of flue gas process treatment stages. Furthermore, the trend of CO_2_ and CH_4_ adsorption shows that our new crystal could be easily regenerated in an efficient process. A close scrutiny of adsorption results shows that CO_2_ (3.1016 m mol/g) sorption is higher than CH_4_ (2.3467 m mol/g) at temperature 298 K and at pressure of 50 bars, where maximum sorption was expected. This comparison gives clear evidence of a selective behavior of the crystal acidic pyrophosphate Rb_2_Co(H_2_P_2_O_7_)·2H_2_O for CO_2_ and CH_4_ gases at each temperature and pressure. On further reviewing Figs. 3 and 4 single crystal sample shows that the sorptivity of CO_2_ is higher than that of the CH_4_ at 318 K temperatures, additionally, ~ 0.5 bar sorptivity difference between both gases at both the temperatures at its highest measured pressure. However, this is the continuation of our recent published research work ^40^ and comparative plot of present and previously investigated has been included in supporting information as Fig. S5. Previously used material ((NH_4_)_2_ Mg(H_2_P_2_O_7_)_2_·2H_2_O) for CO_2_ and CH_4_ at 50 bar, and 298 K and 318 K values shows that adsorb 3.3858 m mol/g, 2.9657 m mol/g of CO_2_ of (NH_4_)_2_ Mg(H_2_P_2_O_7_)_2_·2H_2_O, respectively, at the same pressure and temperatures 5.3234 m mol/g and 8.2909 m mol/g respectively for CH_4_. The CO_2_ sorption is almost close to the currently investigated sample however CH_4_ sorption values are 3–4 fold higher in comparison with Rb_2_Co(H_2_P_2_O_7_)·2H_2_O at temperatures. Moreover, on comparing CO_2_ sorption data of this work and hydroxy metal carbonates M(CO3)_x_(OH)_y_ (M=Zn, Zn–Mg, Mg, Mg–Cu, Cu, Ni, and Pb) ^45^, found that Rb_2_Co(H_2_P_2_O_7_)·2H_2_O shows higher values 35 bar at 318 K, although hydroxy metal carbonates were measured at 316 K. The enhanced CO2 sorption capacity of Rb_2_Co(H_2_P_2_O_7_)·2H_2_O compared to hydroxy metal carbonates may be attributed to its unique three-dimensional framework. The presence of [RbO7], [H2P2O7], and [CoO6] entities interconnected through a dense network of hydrogen bonds can offer specific adsorption sites and channels, facilitating better gas uptake. Furthermore, the preferential adsorption of CO2 in Rb_2_Co(H_2_P_2_O_7_)·2H_2_O over hydroxy metal carbonates can be intricately linked to the electronic nature of its components. The coordination of the Co ion, often known for its variable oxidation state and magnetic properties, combined with the phosphate groups, may provide an electron-rich environment. This can enhance the Lewis acid–base interactions with the electrophilic carbon of CO_2_, resulting in better sorption. Additionally, the dense hydrogen-bonding network might create a dynamic environment, allowing for reversible adsorption and facilitating efficient gas uptake and release. In Fig. 5, we have demonstrated the CO_2_/CH_4_ ideal selectivity results at 298 K and 318 K from pressure 1–50 bar (11 sorption data point). The CO_2_/CH_4_ (mmol gas per g of sample) ideal selectivity at 298 K was higher as compared to 318 K, as expected, and trends of variations are close to each other at both the temperatures. Furthermore, after 35 bar significantly increased values were noticed, and difference was reached at maximum investigated pressure. Last but not the least, CO_2_/CH_4_ ideal selectivity of Rb_2_Co(H_2_P_2_O_7_)·2H_2_O is much higher than (NH_4_)_2_Mg(H_2_P_2_O_7_)_2_·2H_2_O of previous work ^40^ at all the pressures and temperatures. CO_2_, with its linear molecular geometry and inherent quadrupole moment, interacts differently with adsorbents compared to tetrahedral CH_4_. Rb_2_Co(H_2_P_2_O_7_)·2H_2_O might provide specific sites where the CO2 quadrupole can align favorably, leading to enhanced selectivity. Rb’s relatively larger ionic size may influence the pore geometries and accessibility, leading to differential interactions with CO_2_ and CH_4_ molecules, thus affecting the separation efficiency. Furthermore, due to these large ionic radii for Rb and diffuse electron cloud can also lead to induction of more significant dispersion forces. This, combined with the potential electronic interactions from Co and phosphate entities, might selectively favor the adsorption of CO2 over CH4, fine-tuning the separation efficiency.

### Simulation results for CO_2_ and CH_4_ adsorption

**Figure 6:**
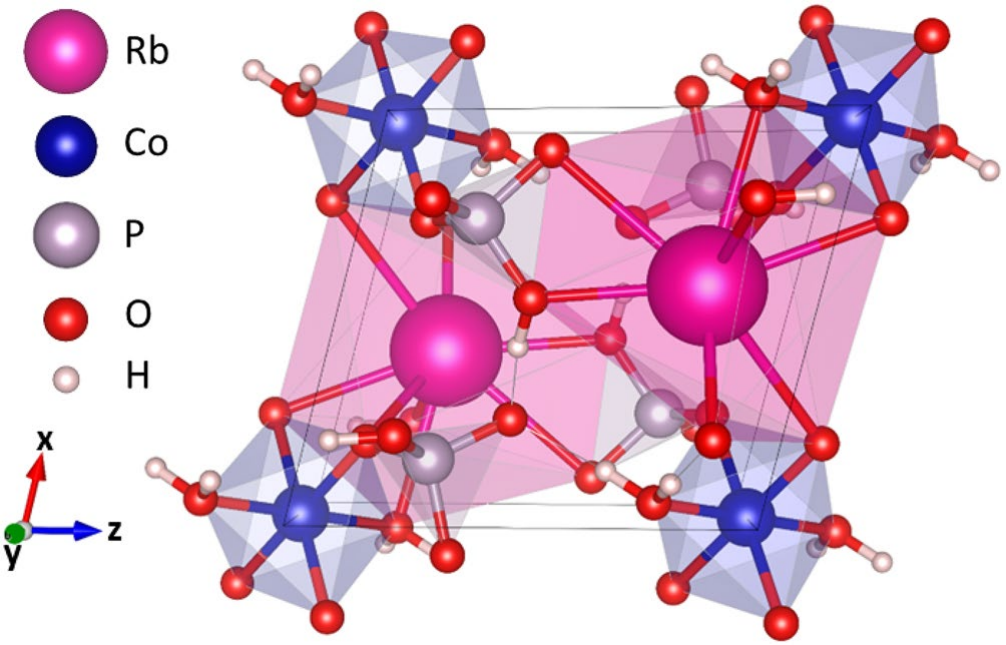
Optimized unit cell structure of Rb_2_Co(H_2_P_2_O_7_)_2_·2H_2_O.

We started by creating and optimizing the unit cell structure of the Rb_2_Co(H_2_P_2_O_7_)·2H_2_O material using our experimental findings. Figure 6 shows the unit cell structure of the material after geometry optimization. The optimized structure has a triclinic lattice with parameters a = 7.008 Å, b = 7.44 Å, and c = 7.71 Å, and angles α = 82.24, β = 72.44 and γ = 86.42. Using this unit cell structure, we have constructed and further optimized 21 slab geometries with 5 different surface symmetries (100, 001, 010, 101, 011, 110, and 111) and 3 different terminations (P-, Ru-, and Co-terminations). Total energy calculations^46,47^ show that a slab with Co-terminated 111 surface has the lowest energy for this material. Next, we study the adsorption of CO_2_ and CH_4_ molecules on this surface. To characterize the adsorption properties of the material, we have conducted total energy and electron localization function calculations. The adsorption energies are calculated as:

$${E}_{ads}={E}_{(slab+mol)}-{E}_{slab}-{E}_{mol},$$

where E_mol_ is the total energy of the isolated molecule and E_slab_ is the total energy of the slab in the absence of the molecule.

**Figure 7:**
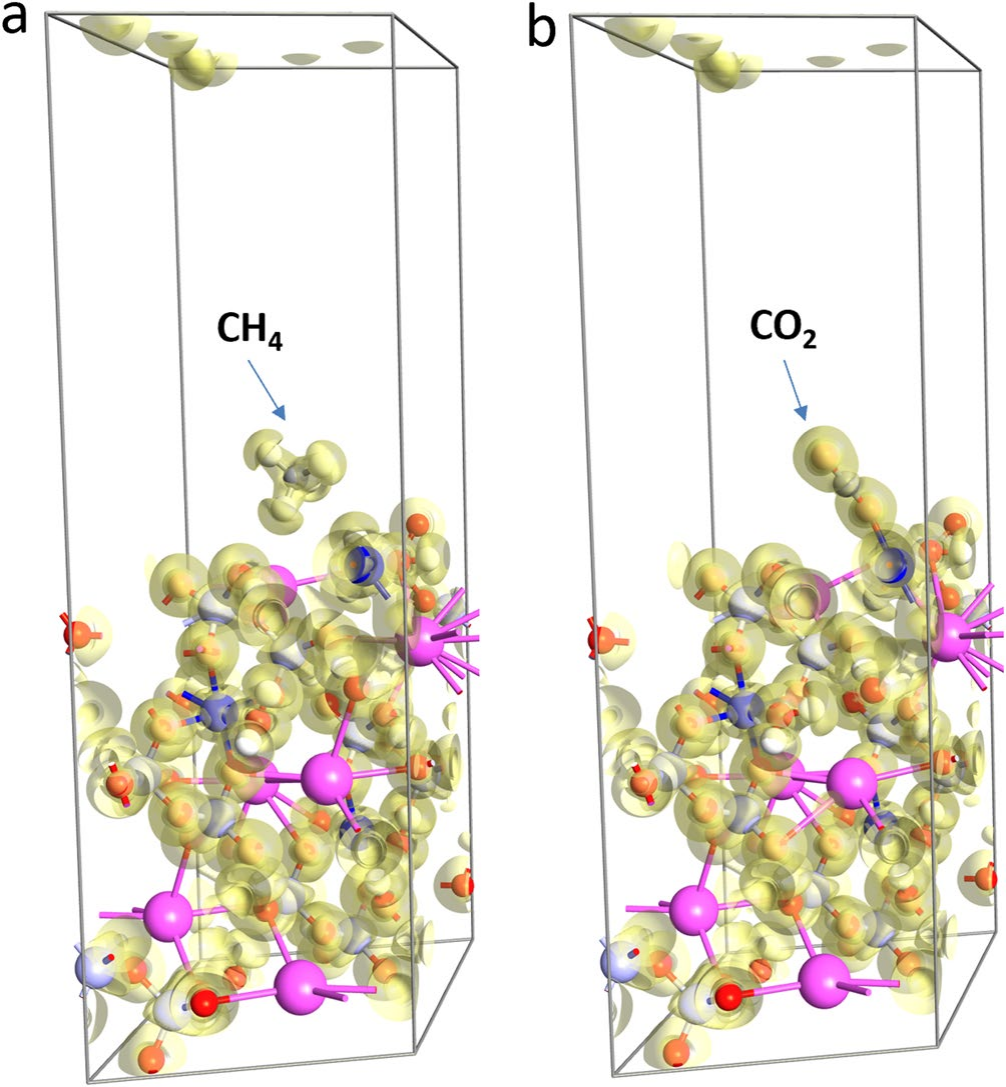
Lowest energy configurations for CH_4_ (**a**) and CO_2_ (**b**) molecules on Co-terminated 111 surface of Rb_2_Co(H_2_P_2_O_7_)_2_·2H_2_O. The figure also shows the isosurface plots (isovalue ± 0.1 Å^−3^) of the electron difference density.

To find the adsorption sites with minimum energy, we have started our calculations for 15 different locations and orientations of the molecules on Co-terminated 111 surface of Rb_2_Co(H_2_P_2_O_7_)·2H_2_O. The atoms at bottom layer of the slab kept fixed during structural optimization to represent the bulk of the material. Figure 7 shows the lowest energy configurations for CH_4_ (a) and CO_2_ (b) molecules. CH_4_ molecule is physisorbed on the surface of the slab near the Co atom and therefore remains unperturbed (see Fig. 7a). The calculated adsorption energy of this molecule is − 0.42 eV. CO_2_ molecule is adsorbed more strongly to the surface with adsorption energy of − 0.93 eV. The electronic structure analysis shows the covalent bond formation between the molecule and Co atom of the material (Fig. 7b). Indeed, we obtained a strong overlap of the electron localization function between the molecule and the substrate. Thus, DFT calculations also reveal strong gas adsorption properties of the synthesized Rb_2_Co(H_2_P_2_O_7_)·2H_2_O material.

## Conclusions

The new pyrophosphate Rb_2_Co(H_2_P_2_O_7_)·2H_2_O compound was successfully synthesized in a solution-like and was found to crystallize in the triclinic system, space group ($P\overline{1}$). Its UV–Vis spectrum has shown the usual transitions between the ground state 4T1g and the supper levels 4T2g, 4A2g and 4T1g(P). The sorption results clearly revealed that the prepared compound manifested a high adsorption capacity of the CO_2_ and CH_4_ gases at different pressures and temperatures. By increasing the temperature, the sorption capacity decreased and increases with respect to the pressure, leading to high sorption values which are quite normal thermodynamically. However, a common adsorption/desorption track of pressure trends alludes to the physisorption phenomena. More interestingly, gas sorption comparison study showed clear evidence of a selective behavior of the acidic pyrophosphate Rb_2_Co(H_2_P_2_O_7_)·2H_2_O crystal for CO_2_ and CH_4_ gases at each temperature and pressure, and revealed also that the sample adsorbtivity of CO_2_ is more than that of the CH_4_ at different temperatures and pressures. Computer simulations also reveal the selectivity of the material to CO_2_ molecules as compared to methane molecules.

### Supplementary Information

Supplementary Information.

## Data Availability

Supplementary tables of crystal structures and refinements, notably full bond lengths and angles, and anisotropic thermal parameters have been deposited with the Inorganic Crystal Structure Database, FIZ, Hermann von Helmholtz Platz 1, 76344 Eggenstein Leopoldshafen, Germany; fax: (+ 49) 7247 808 132; Email: crysdata@fiz-karlsruhe.de. CSD-deposition numbers are respectively 421807 for Rb_2_Co(H_2_P_2_O_7_)·2H_2_O.
