# Supplementary material for: Efficient crystal structure materials as reactive sorbent for the CO2 and CH4 adsorption and storage
Source: Sci Rep. 2024 Mar 19;14:6599. doi: 10.1038/s41598-024-57060-8 (PMC10951319; doi:10.1038/s41598-024-57060-8)
Supplement: Supplementary file 1 — Supplementary Information. [file 41598_2024_57060_MOESM1_ESM.doc]

**SUPPORTING INFORMATION**

**Efficient crystal structure materials as reactive sorbent for the CO_2_ and CH_4_ adsorption and storage**

R. Essehli^1^, B. Aïssa^2^, T. Altamash^2,3^, M. Lachkar^4^, M. Atilhan^5^, B. El Bali^6^, G. R. Berdiyorov^2^,

A. Amhamed^*2^

^1^Energy and Transportation Science Division, Oak Ridge National Laboratory (ORNL), 1 Bethel Valley Rd, Oak Ridge, TN 37830, USA.

^2^Qatar Environment & Energy Research institute (QEERI), Hamad Bin Khalifa University (HBKU), Qatar Foundation, P.O. Box 34110 Doha, Qatar.

^3^Materials Science, Energy and Nanoengineering Department (MSN), Mohammed VI Polytechnic University (UM6P), Lot 660 – Hay Moulay Rachid, 43150, Ben Guerir, Morocco

^4^University Sidi Mohamed Ben Abdellah, Fez city, Morocco

^5^Western Michigan University, Department of Chemical and Paper Engineering, Floyd Hall, A-230, 49008, Kalamazoo, MI, USA.

^6^Laboratory of Mineral Solid and Analytical Chemistry, ‘’LMSAC’’, Department of Chemistry,

Faculty of Sciences, University Mohamed I, Po. Box 717, 60000 Oujda, Morocco

* Corresponding authors: A. Amhamed (aamhamed@hbku.edu.qa).

**Figure S1:** CO_2_ adsorption-desorption trend of variation at 298 K

**Figure S2:** CO_2_ absorption-desorption trend of variation at 318 K

**Figure S3:** CH_4_ absorption-desorption trend of variation at 298 K

**Figure S4:** CH_4_ absorption-desorption trend of variation at 318 K


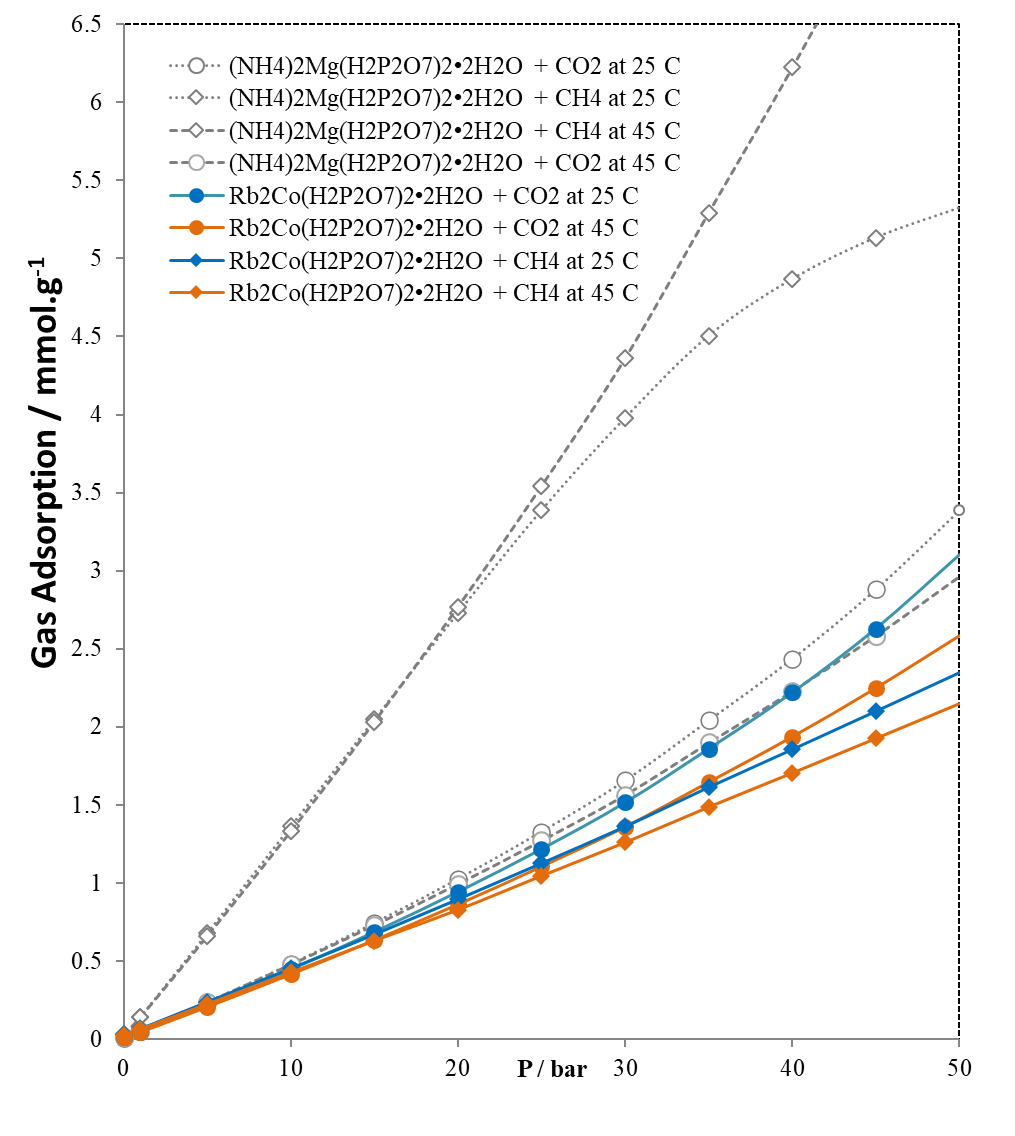


**Figure S5:** A comparative study of CO_2_ and CH_4_ sorption trend of variation at temperate 298 and 318 K, and at all invetigated pressures.

**Table S1: CO_2_ adsorption values in mmol/g of sample (Ads.=Adsorption; Des.=Desorption) at different pressures and temperatures.**

| **T = 298 °C** | | | | **T = 318 °C** | | | | |
| --- | --- | --- | --- | --- | --- | --- | --- | --- |
| **P/ bar** | **mmol/g CO_2_ (Ads.)** | **P/ bar** | **mmol/g CO_2_ (Des.)** | **P/ bar** | **mmol/g CO_2_ (Ads.)** | **P/ bar** | **mmol/g CO_2_ (Des.)** | |
| 0.05 | 0.0128 | 40.00 | 2.2348 | 0.05 | 0.0129 | 40.00 | 1.9440 | |
| 1.00 | 0.0493 | 30.00 | 1.5266 | 1.00 | 0.0480 | 30.00 | 1.3703 | |
| 5.00 | 0.2221 | 20.00 | 0.9543 | 5.00 | 0.2093 | 20.00 | 0.8725 | |
| 10.00 | 0.4495 | 10.00 | 0.4569 | 10.00 | 0.4180 | 10.00 | 0.4238 | |
| 15.00 | 0.6888 | 5.00 | 0.2292 | 15.00 | 0.6362 | 5.00 | 0.2141 | |
| 20.00 | 0.9454 | 1.00 | 0.0723 | 20.00 | 0.8649 | 1.00 | 0.0665 | |
| 25.00 | 1.2190 | 0.05 | 0.0138 | 25.00 | 1.1066 | 0.05 | 0.0131 | |
| 30.00 | 1.5169 |  |  | 30.00 | 1.3605 |  |  | |
| 35.00 | 1.8609 |  |  | 35.00 | 1.6468 |  |  | |
| 40.00 | 2.2207 |  |  | 40.00 | 1.9363 |  |  | |
| 45.00 | 2.6299 |  |  | 45.00 | 2.2483 |  |  | |
| 50.00 | 3.1016 |  |  | 50.00 | 2.5827 |  |  | |
| **Table S2:** CH4 adsorption values in mmol/g of sample (Ads.=Adsorption; Des.=Desorption) at different pressures and temperatures. | | | | | | | | |
| **T = 298 °C** | | | | **T = 318 °C** | | | | |
| **P/ bar** | **mmol/g CH_4_ (Ads.)** | **P/ bar** | **mmol/g CH_4_ (Des.)** | **P/ bar** | **mmol/g CH_4_ (Ads.)** | **P/ bar** | | **mmol/g CH_4_ (Des.)** |
| 0.05 | 0.0334 | 40.00 | 1.8562 | 0.05 | 0.0301 | 40.00 | | 1.7095 |
| 1.00 | 0.0707 | 30.00 | 1.3698 | 1.00 | 0.0655 | 30.00 | | 1.2657 |
| 5.00 | 0.2417 | 20.00 | 0.9103 | 5.00 | 0.2259 | 20.00 | | 0.84467 |
| 10.00 | 0.4561 | 10.00 | 0.4635 | 10.00 | 0.4282 | 10.00 | | 0.43237 |
| 15.00 | 0.6737 | 5.00 | 0.2455 | 15.00 | 0.6309 | 5.00 | | 0.2301 |
| 20.00 | 0.8982 | 1.00 | 0.0785 | 20.00 | 0.8295 | 1.00 | | 0.0696 |
| 25.00 | 1.1284 | 0.05 | 0.0315 | 25.00 | 1.0463 | 0.05 | | 0.0301 |
| 30.00 | 1.3633 |  |  | 30.00 | 1.2604 |  | |  |
| 35.00 | 1.6142 |  |  | 35.00 | 1.4871 |  | |  |
| 40.00 | 1.8561 |  |  | 40.00 | 1.7047 |  | |  |
| 45.00 | 2.1001 |  |  | 45.00 | 1.9258 |  | |  |
| 50.00 | 2.3467 |  |  | 50.00 | 2.1484 |  | |  |

Standard uncertainties u are u(P) = 0.035 bar, u(T) = 0.05 K
